# Prevalence of Hypertension among Adults in Remote Rural Areas of Xinjiang, China

**DOI:** 10.3390/ijerph13060524

**Published:** 2016-05-24

**Authors:** Yulin Wang, Jingyu Zhang, Yusong Ding, Mei Zhang, Jiaming Liu, Jiaolong Ma, Heng Guo, Yizhong Yan, Jia He, Kui Wang, Shugang Li, Rulin Ma, Bek Murat, Shuxia Guo

**Affiliations:** 1 Department of Public Health, School of Medicine, Shihezi University, Beier Road, Shihezi 832000, China; nihaowangyulin@163.com (Y.W.); yfyxxzjy@126.com (J.Z.); 13399931625@163.com (Y.D.); zmberry@foxmail.com (M.Z.); liujiaming@shzu.edu.cn (J.L.); jiaojiaolong881202@163.com (J.M.); guoheng@shzu.edu.cn (H.G.); erniu19880215@sina.com (Y.Y.); hejia123.shihezi@163. Com (J.H.); kwang311@hotmail.com (K.W.); lishugang@ymail.com (S.L.); marulin@126.com (R.M.); murat08123@163.com (B.M.); 2 Department of Pathology and Key Laboratory of Xinjiang Endemic and Ethnic Diseases (Ministry of Education), School of Medicine, Shihezi University, Beier Road, Shihezi 832002, China

**Keywords:** hypertension, prevalence, epidemiological studies, rural, nation

## Abstract

*Objective:* The present study aimed to estimate prevalence of hypertension among adults in rural remote areas of Xinjiang, China and evaluate the associated factors of hypertension. *Methods:* The survey was based on questionnaire interviews and clinical measurements of 11,340 individuals (≥18 years old), and was conducted during 2009–2010 via a stratified cluster random sampling method in the remote rural areas of Xinjiang, about 4407 km away from the capital Beijing. Hypertension was defined according to WHO/ISH criteria. *Results:* Systolic blood pressure (SBP) and diastolic blood pressure (DBP) of the population were (126.3 ± 21.4) and (80.9 ± 13.4) mmHg. Compared with Han nationality subjects, SBP and DBP of Kazakh nationality subjects were significantly high (*p* < 0.05), while the SBP and DBP of Uyghur subjects were significantly low (Kazakh: (128.7 ± 23.9) and (83.0 ± 14.6) mmHg, Uyghur: (123.6 ± 19.3) and (77.4 ± 12.7) mmHg, Han: (126.5 ± 20.5) and (82.6 ± 11.9) mmHg, *p* < 0.05). Prevalence of hypertension of the population was 32.1%, and was greater among Kazakhs and lower among Uyghur than Han (Kazakh: 36.9%, Uyghur: 26.1%, Han: 33.7%, *p* < 0.05). The age-standardized prevalence of hypertension was 30.2%, and was greater among Kazakhs while lower among Uyghurs than Han subjects (Kazakh: 37.0%, Uyghur: 26.0%, Han: 33.8%, *p* < 0.05, *p* < 0.05). Multivariate logistic regression analyses showed Gender (OR = 1.324), age (OR = 2.098, 3.681, 6.794, 9.473, 14.646), nationality (OR = 1.541), occupation (OR = 1.659, 1.576), education (OR = 1.260), BMI (OR = 1.842), WC (OR = 1.585), WHR (OR = 1.188), WHR (OR = 1.188), diabetes (OR = 1.879), hypertriglyceridemia (OR = 1.361), hypercholesterolemia (OR = 1.131) and high blood low density lipoprotein cholesterol (LDL-C) (OR = 1.956) were all positively correlated with hypertension, while low blood high density lipoprotein cholesterol (HDL-C) (OR = 0.765) was negatively correlated with hypertension. *Conclusions:* Prevalence of hypertension among adults in remote rural areas of Xinjiang was higher than the national average. Prevalence of hypertension was greater among Kazakhs and lower among Uyghurs than Han nationals, thus indicating significant differences between regions and nationalities. Gender, age, nation, occupation, education, overweight or obesity, abdominal obesity, diabetes, hyperlipidemia, hypercholesterolemia, high LDL-C were positively correlated with hypertension, low HDL-C was negatively correlated with hypertension.

## 1. Introduction

Hypertension is the most common chronic disease, and the most important risk factor of cardiovascular diseases. Data has shown that lowering the blood pressure in hypertensive patients can reduce the risks of stroke and heart disease events; it can also improve the life quality of hypertensive patients significantly [1,2]. In China, with a population of 1.34 billion, about 200 million people suffer from hypertension [3]. In other words, there were two people suffering from hypertension for every ten adults in China, accounting for about 1/5 of the global number of hypertensive patients [1], China had become the first hypertensive country around the world. Xinjiang, a vast and multi-ethnic province, is located on the northwest edge of China; its prevalence of hypertension ranks at the forefront of China due to its geographical remoteness, a relatively backward economy and low education level. The rates of awareness, treatment and control of hypertension are very low, and moreover, hypertension in remote rural areas of Xinjiang was even more worrying [4,5,6]. Despite China’s economic growth, social progress, improved living standards and accelerated urbanization, especially in economically backward areas, such as Xinjiang, there have been only a few epidemiological investigations of hypertension among urban populations. Xinjiang has a broadly dispersed population, and it is a multinational region with forty-seven nationalities. The main ethnic groups in Xinjiang are the Uygur, Kazak and Han. There are serious language communication barriers in the epidemiological investigation of ethnic minorities, especially in remote rural areas, so studies in remote rural areas are almost non-existent due to the implementation difficulties. Jiashi, Xinyuan and Shawan County were the regions mainly inhabited of the Uygur, Kazak and Han population, respectively. To further explore the epidemiological characteristics of hypertension among the Uygur, Kazak and Han populations in remote rural areas of Xinjiang, and strengthen the level of awareness, prevention and treatment of hypertension, our research team launched an epidemiological investigation among rural adults in Jiashi, Xinyuan and Shawan County, Xinjiang and quantitatively evaluated the effects of different levels of body mass index (BMI) and waistline circumference (WC) on the prevalence of hypertension.

## 2. Subjects and Methods

### 2.1. Subjects and Sampling

This questionnaire-based survey and clinical measurements of 11,340 individuals were conducted during 2009 to 2010, using a multistage stratified cluster random sampling method (region-county-township-village) among rural residents. At the beginning, we chose three representative regions (Kashi, Yili, Tacheng) according to the geographical distribution of the population in Xinjiang (which is located in the northwest in China). After that, we randomly selected three counties (Jiashi, Xinyuan, and Shawan County) from the three regions. Then, three townships (Jiangbazi, Nalati and Xigebi Township) were randomly chosen from the three counties. In the last stage, a stratified sampling method was used to select corresponding villages (twelve in Jiangbazi, six in Nalati, nine in Xigebi) in the three townships. With informed consent, we interviewed permanent residents (*i.e.*, who had been residing for more than 6 months in the village) of the Han, Uyghur and Kazakh nationalities aged 18 and above for investigation. The study was approved by the Ethical Review Board of The First Affiliated Hospital of Medical College of Shihezi University (ID: 2010LL01).

The number of samples in each age group was calculated according to the age composition of the national population census in 2000. Men and women were stratified to ensure gender balance in the sample. Four thousand people were estimated in each sampling region, and a total of 12,000 people were investigated. Finally, the survey was actually completed by 11,340 people actually, so the response rate was 94.5%. The number of Kazakh, Uyghur and Han subjects were 3904, 3976 and 3460, respectively.

### 2.2. Data Collection

Every investigator underwent strict training in the methodology and principles of the research program as well as in the necessary skills for the study prior to the start of the research. Data comprised questionnaire interviews and clinical measurements.

### 2.3. Questionnaire Interviews

A self-developed questionnaire was applied by trained investigators to collect detailed information. The questionnaire consisted of demographic information about the respondents (such as age, gender, nationality, education level, marital status, *etc*.) and personal lifestyles (such as cigarette smoking, alcohol drinking, physical activity, dietary habits, *etc.*).

### 2.4. Clinical Measurements

Blood pressure, body weight, height, and WC were measured by standard methods during the interview and physical examinations [7]. Height was measured to the nearest 0.1 centimeter (cm), without shoes, with the participant’s back square against the wall tape, eyes looking straight ahead with a right-angle triangle resting on the scalp and against the wall. Weight was measured in light undergarments without shoes with a lever balance to the nearest 0.1 kilogram (kg). WC was defined as the midpoint between the lower rib and upper margin of the iliac crest, measured by a ruler tape with an insertion buckle at one end. WC and HC was measured to the nearest 0.1 cm. BMI was calculated by dividing weight (in kilograms) by height (in meters) squared (kg/m^2^). To ensure comparability with other studies, our study incorporated the criteria recommended by the Working Group on Obesity in China (WGOC) (Normal: BMI < 24.0 kg/m^2^; Overweight: BMI 24.0 ≤ 28.0 kg/m^2^; General obesity: BMI ≥ 28 kg/m^2^; Abdominal obesity: WC ≥ 85 cm for men and ≥ 80 cm for women [8] and the WHO classifications for Europids (Normal: BMI < 25.0 kg/m^2^; Overweight: BMI 25.0 ≤ 30.0 kg/m^2^; General obesity: BMI ≥ 30 kg/m^2^; Abdominal obesity: WC ≥ 102 cm for men and ≥88 cm for women) [9]. Blood pressure was measured three times on each subject in a quiet room, using a platform mercuric sphygmomanometer in the sitting position after a 15 min rest. Time interval was greater than 2 min [1].

### 2.5. Diagnostic Criteria

Hypertension was defined as SBP ≥ 140 mmHg or DBP ≥ 90 mmHg, or current use of any antihypertensive medication or any combination of the above [10]. Dyslipidemia was defined according to the prevention standard proposed for dyslipidemia in China in 2007 (Joint Committee for Developing Chinese Guidelines on Prevention and Treatment of Dyslipidemia in Adults, 2007), TG ≥ 2.26 mmol/L, TC ≥ 6.22 mmol/L, LDL-C ≥ 4.14 mmol/L and HDL-C < 1.04 mmol/L were considered indicative of hypertriglyceridemia, hypercholesterolemia, high LDL-C and low HDL-C, respectively [11].

### 2.6. Statistical Analysis

A databank was created using the EpiData software (EpiData Association: Odense, Denmark). Data was analyzed using the Statistical Program for Social Sciences, version 17.0 (SPSS Inc.: Chicago, IL, USA). Continuous variables were presented as means ± standard deviations (m ± sd), and were analyzed using *t*-test and ANOVA. Categorical variables were expressed as proportion (%) and frequency (n), and were analyzed using the Chi-square test or Chi-square trend test. As for hypertension, adjusted odds ratio (OR) with associated 95% confidence, interval (95%CI) were calculated using multivariate logistic regression model, using the enter method. The official 2000 census data of China was used to calculate age-standardized prevalence of hypertension, using the direct method and 10-year ages range. All statistical tests were two-sided and differences were considered statistically significant when the *p* value was < 0.05.

## 3. Results

### 3.1. Basic Characteristics of Population

There were 4962 (43.8%) men and 6378 (56.2%) women among the 11,340 adults. The Kazakh, Uyghur, and Han nationalities accounted for 34.4% (3904), 35.1% (3976) and 30.5% (3460), respectively. Illiterates, primary education and below accounted for 19.4% (2200) and 88.8% (10,070). People with agriculture and animal husbandry occupations accounted for 69.0% (7829). Table 1 showed the age, BMI and WC data of the study population.

### 3.2. Level of Blood Pressure by Age and Nation

Table 2 shows levels of blood pressure based on age and nationality classifications. SBP and DBP of the population were (126.3 ± 21.4) and (80.9 ± 13.4) mmHg. Compared with Han, SBP and DBP of Kazakh were significantly high (*p* < 0.05), while SBP and DBP of Uyghur were significantly low (*p* < 0.05). SBP and DBP increased significantly with the increase in age (*p* < 0.05).

### 3.3. Prevalence of Hypertension by Age and Nationality

Table 3 shows the prevalence of hypertension based on age and nationality classifications. Prevalence of hypertension of the population was 32.1%. Prevalence of hypertension was greater among Kazakhs and lower among the Uyghur than the Han subjects (*p* < 0.05). Prevalence of hypertension increased significantly with the increase in age (*p* < 0.05).

### 3.4. Prevalence of Hypertension by BMI and Nationality

Table 4 shows the prevalence of hypertension increased significantly with the increase in BMI (*p* < 0.05). Prevalence of hypertension was greater among Kazakhs and lower among Uyghur than Han in each BMI group (*p* < 0.05).

### 3.5. Prevalence of Hypertension by WC and Nation

Table 5 shows the prevalence of hypertension increased significantly with the increase in WC (*p* < 0.05). Prevalence of hypertension was greater among Kazakh nationals and lower among Uyghur than Han in each WC group (*p* < 0.05).

### 3.6. Adjusted ORs of Associated Factors for Hypertension

A multivariate logistic-regression analysis, using the enter method, was used to determine the adjusted odds ratios (ORs) of the independent predictors of hypertension. A *p* value of < 0.05 was considered statistically significant. The results of multivariate logistic-regression analyses showed that hypertension was statistically significantly associated with gender, age, nation, BMI, WC, WHR, diabetes, hyperlipidemia, high cholesterol, high LDL-C and low LDL-C (*p* < 0.05, Table 6).

## 4. Discussion

Hypertension remains a major health and economic problem around the world today [12]. It is related to many factors, and there are significant differences between regions and nationalities. This survey found that the standardized prevalence of hypertension in remote rural areas of Xinjiang was 30.2%, and 37.0%, 26.0% and 27.8% in the Kazakh, Uyghur and Han population, respectively, which was significantly higher than the results of the China National Nutrition and Health Survey (where the prevalence of hypertension of national and rural areas were 18.8% and 12.6% [13,14]). The underlying reasons may be geographical and policy remoteness, medical care and education disadvantages, unbalanced diet and insufficient physical activities.

Firstly, Xinjiang, occupying a vast territory, is located in the northwest of China and is geographically remote from the central government. The distance from our investigation areas to the capital Beijing is about 4407 km, and the investigation areas were the most remote areas of China, so it was difficult for national policies and systems to reach the region. By 2014, Xinjiang’s GDP ranked 25th among 31 provinces in China, and lagged behind the whole national level. For example, in Jiashi County, an Uyghur concentrated areas, more than 92% of Uyghurs live on US $1.00 per day or less, in sharp contrast to the national average whereby 15.9% of people live on US $1.00 per day [15,16].

Secondly, people with agriculture and animal husbandry occupations accounted for 69.0% (7829/11,340) in the total study population. Besides, the survey population had a low level of education, so the illiteracy rate was 19.4%, higher than the national illiteracy rate of 4.1%, and primary education and below accounted for 88.8%, higher than the national rate of 64.3% [17]. Epidemiological studies carried out in the United States, Britain, Norway and other countries have shown a significant negative correlation between education level and blood pressure [18,19,20,21]. Due to their geographical remoteness and economic backwardness, the rural areas of Xinjiang are extremely backward in medical conditions. Low education level, poor health awareness, and unhealthy life styles among its ethnic minorities make the prevalence of hypertension, disability and mortality rate be at high levels in the remote rural areas of Xinjiang.

Thirdly, Xinjiang was a minority-populated area, Uyghur and Kazakh are the two main ethnic minorities, and their living environment and bad eating habits led to a higher prevalence of hypertension than national the national average [3,5]. Kazakhs and Uyghurs accounted for 34.4% and 35.1% of the survey population, respectively, causing the prevalence of hypertension in the remote rural areas of Xinjiang to be at a high level.

Epidemiological studies have shown with an average dietary salt intake increase of 2 g/d, SBP and DBP respectively increased by 2.0 and 1.2 mmHg [22]. The daily salt intake of Kazakhs is high. Their staple food and drink are mainly the salty roasted nang and salty milk tea, resulting in a salt intake >40 g/d, while the average salt intake in most parts of China is only 12 g/d [23]. The daily foods were mainly pasta, beef and mutton, dairy foods (high in animal fat) and bacon-based products, but fresh vegetables and fruits were scarce. This unique diet may cause obesity, diabetes, dyslipidemia, etc., which can affect their blood pressure level.

The survey population was mainly farmers and herdsmen, with poor economic status and education. Specifically the basic diet of the Uyghur contains carbohydrates and proteins but was deficient in vitamins, so obesity among the Uyghur is serious, which makes their blood pressure high.

Lastly, Xinjiang is located in the northwest inland area of China, where rural people do not need to engage in agricultural production during the long and cold winter. For example, the Kazakh are nomadic and reside in mountainous areas where outdoor activities are impractical during the cold winter, and the few outdoor activities increases their risk of hypertension.

These factors mention above cause obesity, diabetes, dyslipidemia and other hypertension risk factors gathered together, can lead to serious hypertension, consistent with literature reports [24,25,26,27,28]. In this study, prevalence of hypertension was greater among the Kazakh subjects and lower among the Uyghur than among the Han, and multivariate regression logistic analysis showed that Kazakh showed OR of hypertension while Uyghur and the Han showed reduced OR, which further proved that ethnic and regional differences caused diversity in the prevalence of hypertension [4]. The information above reminds us that further studies on the genetic and environmental interaction mechanism of hypertension, and strength of the relationships should be conducted.

Many studies have confirmed that body fat percentage was positively correlated with blood pressure. Compared with the normal BMI, a BMI ≥ 24 bkg/m^2^ causes three to four times the risk of hypertension, WC ≥ 90 (men) or ≥85 cm (women) cause more than four times of the risk of hypertension than the normal WC [1]. This study found that with increased BMI and WC, the prevalence of hypertension showed an increasing trend. In addition, multivariate analysis showed that the correlation with hypertension was higher among overweight/obesity (OR = 1.842) than abdominal obesity (OR = 1.585), which indicated that maintaining a normal BMI, compared with WC and WHR, may be more significant for the prevention and treatment of hypertension, and general obesity, compared with abdominal obesity, may represent a greater risk of hypertension. Besides, high BMI increased almost 5.5 times the OR of hypertension to the maximum extent, which suggested that obesity maybe an essential risk factor of hypertension among ethnic minorities in the remote rural areas of Xinjiang.

Because the Kazakh and Uyghur reside at the borders where Asian and Caucasian people were mixed together [25], molecular genetics studies have found that the Uyghur have a mixture of 60% European ancestry and 40% East Asian ancestry [29]. This discovery may explain, at least in part, how the genetic influence on the Kazakh and Uyghur caused their obesity rate to be higher than among Chinese, but lower than Europeans. Related research found that prevalence of obesity among the Kazakh and Uyghur was higher than the national average [30], which also explained one of the main reasons why the prevalence of hypertension in the remote rural areas of Xinjiang was so high [31].

Further research should be conducted to explore the environmental and genetic interactions of hypertension. There is an urgent need to undertake effective and comprehensive preventive measures and interventions for hypertension, screening high-hypertension risk groups, establishing scientific and healthy lifestyles, strengthening health investments and policy support in the remote rural areas of Xinjiang, correctly guiding healthy living behavior and eating habits, especially among the minorities, improving the prevention and treatment of hypertension, reducing hypertension and related diseases, which can reduce the risk of cardiovascular disease and promote health.

## 5. Conclusions

The prevalence of hypertension among adults in the rural remote areas of Xinjiang was higher than the national average. Prevalence of hypertension was greater among the Kazakh minority but lower among the Uyghur than Han nationals, and there were significant differences between regions and nationalities. Gender, age, nation, occupation, education, overweight or obesity, abdominal obesity, diabetes, hyperlipidemia, hypercholesterolemia, high LDL-C were positively correlated with hypertension, while low HDL-C was negatively correlated with hypertension.

## Figures and Tables

**Table 1:** Age, BMI, WC and WHR of population.

Characteristics	Nationality					Hypertension				Total
	Kazakh	Uyghur	Han	*F*	*p Value*	Yes	No	*F*	*p Value*	
Age	44.2 ± 13.3	42.5 ± 16.5	49.7 ± 12.4	256.210	<0.001	52.0 ± 13.4	42.1 ± 14.0	−35.975	<0.001	45.3 ± 14.6
BMI	24.3 ± 4.6	22.8 ± 3.4	24.6 ± 3.4	237.903	<0.001	25.3 ± 4.2	23.3 ± 3.6	−25.688	<0.001	23.9 ± 3.9
WC	85.7 ± 12.3	83.9 ± 9.8	84.1 ± 9.9	34.304	<0.001	88.7 ± 11.4	82.7 ± 9.9	−22.738	<0.001	84.6 ± 10.8

**Table 2:** Level of blood pressure by age and nationality.

Age	SBP			*F*	*p Value*	DBP			*F*	*p Value*	Total	
	Kazakh	Uyghur	Han			Kazakh	Uyghur	Han			SBP	DBP
18~	114.4 ± 14.4	113.6 ± 13.2	112.7 ± 12.2	0.583	0.558	72.8 ± 10.9	71.3 ± 10.5	75.8 ± 10.0	5.886	0.003	113.8 ± 13.5	72.0 ± 10.6
25~	118.2 ± 15.8	116.5 ± 13.8	117.2 ± 15.4	2.282	0.102	77.3 ± 11.6	74.2 ± 11.1	79.1 ± 11.0	28.032	<0.001	117.3 ± 14.9	76.2 ± 11.5
35~	122.9 ± 18.1	120.6 ± 15.9	120.7 ± 15.6	5.690	0.003	81.0 ± 12.9	77.4 ± 11.9	81.6 ± 11.4	31.679	<0.001	121.4 ± 16.6	80.0 ± 12.3
45~	132.6 ± 23.0	127.2 ± 19.3	124.1 ± 18.2	40.991	<0.001	86.4 ± 14.3	79.8 ± 12.5	83.0 ± 12.0	48.680	<0.001	128.2 ± 20.9	83.6 ± 13.3
55~	141.1 ± 28.3	134.0 ± 20.7	131.9 ± 22.6	28.039	<0.001	88.7 ± 16.0	82.2 ± 13.5	84.0 ± 12.0	38.392	<0.001	135.5 ± 24.5	85.1 ± 14.0
65~	149.4 ± 29.0	138.9 ± 22.4	141.0 ± 22.4	16.272	<0.001	89.4 ± 15.5	82.9 ± 13.4	84.4 ± 12.0	20.044	<0.001	142.0 ± 24.3	84.9 ± 13.6
*F*	168.785	194.104	108.194	-	-	108.313	86.755	15.670	-	-	30.621	17.430
*p value*	<0.001	<0.001	<0.001	-	-	<0.001	<0.001	<0.001	-	-	<0.001	<0.001
Total	128.7 ± 23.9	123.6 ± 19.3	126.5 ± 20.5	56.444	<0.001	83.0 ± 14.6	77.4 ± 12.7	82.6 ± 11.9	218.041	<0.001	126.3 ± 21.4	80.9 ± 13.4

**Table 3:** Prevalence of hypertension by age and nation.

Age	Kazakh	Uyghur	Han	*χ*^2^	*p Value*	Total
18~	11.1 (32/288)	6.8 (44/646)	12.3 (7/57)	6.001	0.050	8.4 (83/991)
25~	17.6 (124/706)	13.6 (114/837)	19.2 (63/328)	7.279	0.026	16.1 (301/1871)
35~	27.0 (268/994)	21.1 (183/868)	26.2 (234/892)	9.876	0.007	24.9 (685/2754)
45~	47.8 (471/985)	32.1 (194/605)	31.0 (272/878)	67.726	<0.001	38.0 (937/2468)
55~	55.0 (370/673)	44.7 (250/559)	40.3 (341/846)	33.165	<0.001	46.2 (961/2078)
65~	67.8 (173/255)	54.9 (253/461)	54.3 (251/462)	14.355	0.001	57.5 (677/1178)
*χ*^2^*_trend_*	468.925	505.404	163.555	-	-	1110.974
*p value*	<0.001	<0.001	<0.001	-	-	<0.001
Total	36.9 (1438/3901)	26.1 (1038/3976)	33.7 (1168/3463)	110.261	<0.001	32.1 (3644/11340)
Age-standardized prevalence	37.0	26.0	33.8	209.564	<0.001	30.2

**Table 4:** Prevalence of hypertension by BMI and nation.

Nationality	BMI(WGOC)					BMI(WHO)				
	Normal	Overweight	General Obesity	*χ*^2^*_trend_*	*p Value*	Normal	Overweight	General Obesity	*χ*^2^*_trend_*	*p Value*
Kazakh	26.9 (568/2113)	41.2 (442/1073)	59.9 (428/715)	260.052	<0.001	28.4 (695/2444)	45.6 (486/1065)	65.6 (257/392)	247.930	<0.001
Uyghur	22.9 (627/2740)	30.6 (285/930)	41.2 (126/306)	60.195	<0.001	23.1 (713/3085)	34.0 (253/745)	49.3 (72/146)	78.065	<0.001
Han	22.7 (352/1551)	38.7 (542/1400)	53.5 (274/512)	189.550	<0.001	24.5 (487/1988)	43.7 (546/1250)	60.0 (135/225)	200.145	<0.001
*χ*^2^	12.795	25.764	30.192	-	-	21.238	26.993	11.870	-	-
*p value*	0.002	<0.001	<0.001	-	-	<0.001	<0.001	<0.001	-	-
Total	24.2 (1547/6404)	37.3 (1269/3403)	54.0 (828/1533)	564.819	<0.001	25.2 (1895/7517)	42.0 (1285/3060)	60.8 (464/763)	589.426	<0.001

**Table 5:** Prevalence of hypertension by WC and nation.

Nationality	WC(WGOC)				WC(WHO)			
	Normal	Abdominal Obesity	*χ*^2^	*p Value*	Normal	Abdominal Obesity	*χ*^2^	*p Value*
Kazakh	23.4 (363/1553)	45.8 (1075/2348)	201.692	<0.001	30.4 (859/2826)	53.9 (579/1075)	184.223	<0.001
Uyghur	21.1 (381/1802)	30.2 (657/2174)	42.088	<0.001	24.1 (787/3272)	35.7 (251/704)	40.417	<0.001
Han	20.2 (279/1383)	42.7 (889/2080)	189.258		30.0 (855/2853)	51.3 (313/610)	102.417	<0.001
*χ*^2^	4.771	125.934	-	-	38.858	60.460	-	-
*p value*	0.092	<0.001	-	-	<0.001	<0.001	-	-
Total	21.6 (1023/4738)	39.7 (2621/6602)	414.777	<0.001	27.9 (25.1/8951)	47.8 (1143/2389)	342.536	<0.001

**Table 6:** Factors associated with the prevalence of hypertension from multivariate logistic regression.

Variables	Stratification	*β*	*SE*	*χ*^2^	*p Value*	*OR*	*OR* 95%*CI*
Gender	women					1.000	
	men	0.292	0.040	52.227	<0.001	1.339	(1.237, 1.450)
Age	18~					1.000	
	25~	0.741	0.131	32.066	<0.001	2.097	(1.623, 2.710)
	35~	1.287	0.123	109.747	<0.001	3.622	(2.847, 4.608)
	45~	1.901	0.122	243.133	<0.001	6.695	(5.272, 8.503)
	55~	2.242	0.123	333.201	<0.001	9.412	(7.398, 11.974)
	65~	2.693	0.129	436.440	<0.001	14.783	(11.482, 19.033)
Nationality	Han					1.000	
	Kazakh	0.137	0.049	7.878	0.005	1.147	(1.042, 1.263)
	Uyghur	−0.365	0.051	51.324	<0.001	0.694	(0.628, 0.767)
Occupation	worker					1.000	
	farmer	0.506	0.200	6.427	0.011	1.659	(1.122, 2.453)
	herdsman	0.455	0.126	13.110	<0.001	1.576	(1.232, 2.016)
Education	High school or above					1.000	
	primary education or below	0.231	0.062	14.033	<0.001	1.260	(1.117, 1.422)
BMI(WGOC)	normal					1.000	
	overweight	0.624	0.046	184.830	<0.001	1.867	(1.706, 2.043)
	obesity	1.305	0.059	489.529	<0.001	3.687	(3.285, 4.139)
BMI(WHO)	normal					1.000	
	overweight	0.764	0.045	285.442	<0.001	2.148	(1.966, 2.347)
	obesity	1.527	0.079	375.724	<0.001	4.604	(3.945, 5.373)
WC(WGOC)	normal					1.000	
	abdominal obesity	0.872	0.043	404.270	<0.001	2.391	(2.196, 2.603)
WC(WHO)	normal					1.000	
	abdominal obesity	0.861	0.047	332.166	<0.001	2.366	(2.157, 2.595)
WHR	normal					1.000	
	abdominal obesity	0.591	0.042	197.928	<0.001	1.805	(1.663, 1.960)
Diabetes	no					1.000	
	yes	0.631	0.056	127.963	<0.001	1.879	(1.685, 2.096)
Hypertriglyceridemia	no					1.000	
	yes	0.308	0.056	30.077	<0.001	1.361	(1.219, 1.520)
Hypercholesterolemia	no					1.000	
	yes	0.358	0.103	12.201	<0.001	1.131	(1.170, 1.750)
High LDL-C	no					1.000	
	yes	0.671	0.141	22.77	<0.001	1.956	(1.485, 2.577)
Low HDL-C	no					1.000	
	yes	-0.268	0.054	24.877	<0.001	0.765	(0.688, 0.850)
